# A Novel Respiratory Syncytial Virus (RSV) F Subunit Vaccine Adjuvanted with GLA-SE Elicits Robust Protective T_H_1-Type Humoral and Cellular Immunity In Rodent Models

**DOI:** 10.1371/journal.pone.0119509

**Published:** 2015-03-20

**Authors:** Stacie L. Lambert, Shahin Aslam, Elizabeth Stillman, Mia MacPhail, Christine Nelson, Bodrey Ro, Rosemary Sweetwood, Yuk Man Lei, Jennifer C. Woo, Roderick S. Tang

**Affiliations:** Department of Research, MedImmune, Mountain View, California, United States of America; University of Iowa, UNITED STATES

## Abstract

**Background:**

Illness associated with Respiratory Syncytial Virus (RSV) remains an unmet medical need in both full-term infants and older adults. The fusion glycoprotein (F) of RSV, which plays a key role in RSV infection and is a target of neutralizing antibodies, is an attractive vaccine target for inducing RSV-specific immunity.

**Methodology and Principal Findings:**

BALB/c mice and cotton rats, two well-characterized rodent models of RSV infection, were used to evaluate the immunogenicity of intramuscularly administered RSV vaccine candidates consisting of purified soluble F (sF) protein formulated with TLR4 agonist glucopyranosyl lipid A (GLA), stable emulsion (SE), GLA-SE, or alum adjuvants. Protection from RSV challenge, serum RSV neutralizing responses, and anti-F IgG responses were induced by all of the tested adjuvanted RSV sF vaccine formulations. However, only RSV sF + GLA-SE induced robust F-specific T_H_1-biased humoral and cellular responses. In mice, these F-specific cellular responses include both CD4 and CD8 T cells, with F-specific polyfunctional CD8 T cells that traffic to the mouse lung following RSV challenge. This RSV sF + GLA-SE vaccine formulation can also induce robust RSV neutralizing titers and prime IFNγ-producing T cell responses in Sprague Dawley rats.

**Conclusions/Significance:**

These studies indicate that a protein subunit vaccine consisting of RSV sF + GLA-SE can induce robust neutralizing antibody and T cell responses to RSV, enhancing viral clearance via a T_H_1 immune-mediated mechanism. This vaccine may benefit older populations at risk for RSV disease.

## Introduction

Respiratory syncytial virus (RSV) causes significant respiratory disease burden in young children, immunocompromised patients and elderly individuals [1]. In these populations RSV re-infections can cause respiratory diseases including bronchiolitis and pneumonia, sometimes requiring hospitalization. Despite the medical and economic significance of RSV infections, no vaccine is currently approved for human use [2].

RSV immunity that develops naturally as a result of infection includes both humoral and cellular immune responses, though these responses are insufficient to block re-infections that occur throughout life [3]. RSV infection induces neutralizing antibodies that target the RSV fusion (F) and attachment (G) envelope glycoproteins [2]. These neutralizing antibodies play a significant role in RSV immunity, providing protection upon passive transfer [4, 5] and decreasing the risk of severe RSV disease [6, 7]. F-directed neutralization responses are particularly desirable as F glycoprotein is highly conserved between the RSV A and RSV B viral strains and essential to viral entry [8]. Cellular responses to RSV are also believed to play a role in disease protection. The F glycoprotein contains multiple mouse and human CD4 and CD8 T cell epitopes [9]. RSV-specific CD4 T cell responses promote both B cell antibody production and CD8 responses, with T_H_1-type CD4 responses promoting CD8 responses more effectively than T_H_2-type responses [2]. RSV-specific CD8 T cell responses are detected in seropositive human adults [10, 11] and in the absence of antibody responses can clear virus-infected cells and resolve RSV infection in animal models [12–14]. The role of RSV-specific CD8 T cell responses in resolving RSV disease in humans is less clear.

The balance of RSV-specific antibodies and cellular immunity required to protect against RSV disease in humans is not well understood and may vary with different age groups. RSV re-infections in older adults can result in severe RSV disease despite the presence of robust serum RSV neutralizing titers [7]. In older adults, cellular responses are in general more T_H_2-biased and wane more rapidly than in young adults [15]. RSV-specific cellular responses are reportedly more T_H_2-biased in older adults than in younger adults [10, 11]. Additionally, RSV-specific CD8 T cell responses are weaker in RSV disease-susceptible older adults than in RSV disease-resistant healthy young adults [10, 11]. These observations suggest that an effective RSV vaccine for older adults may need to boost both RSV-specific neutralizing antibodies and T_H_1-biased cellular immunity.

Several adjuvant formulations capable of stimulating both humoral and cellular immunity have been investigated in the context of experimental RSV vaccines in animals. Novel adjuvant compounds incorporating Toll-like receptor (TLR)9 agonists have been shown to improve T_H_1-biased cellular responses to RSV vaccines in mouse models [16–18]. TLR4-based adjuvants such as a Monophosphoryl Lipid A (MPL)/QS-21 combination or Protollin, a formulation of LPS complexed with meningococcal outer membrane proteins, have also been able to induce cellular IFNγ production to RSV vaccines in mice [19, 20]. The TLR4 agonist glucopyranosyl lipid A (GLA) is a recently developed synthetic hexaacylated lipid A derivative demonstrated to be a potent T_H_1-biasing adjuvant in both rodent and primate model systems [21, 22]. GLA can easily be formulated with protein vaccines either alone or in a squalene-based oil-in-water stable emulsion (SE).

In the present study, we used naive adult BALB/c mice and cotton rats to evaluate the immunogenicity of RSV soluble F (sF) subunit protein vaccines formulated with three novel adjuvants not previously tested with RSV proteins. The best formulation was then evaluated in Sprague Dawley rats to characterize immunogenicity at the anticipated clinical antigen doses. Purified sF proteins were formulated with GLA, SE, or GLA-SE and compared in vaccine performance to sF formulated with alum or left unadjuvanted. The results demonstrate that while each intramuscularly-administered adjuvanted RSV sF vaccine formulation induces RSV neutralizing titers and confers protective immunity against viral replication, only RSV sF + GLA-SE vaccines prime IFNγ-producing T cell responses in both BALB/c and cotton rat models. In the BALB/c mouse, these T cell responses are primarily CD8+, can traffic to the lung, and correlate with a T_H_1-biased cytokine response. In the cotton rat, the T cell responses and neutralizing responses observed with RSV sF + GLA-SE correlated with complete protection in the lungs as well as in the nose. In Sprague Dawley rats, 10 to 100 μg of RSV sF + GLA-SE induced robust RSV neutralizing titers and primed IFNγ-producing T cell responses. Overall, the immunogenicity profile of RSV sF + GLA-SE in naïve rodent models suggests that it could potentially boost both RSV neutralizing titers and T-cell immunity in primates.

## Materials & Methods

### Vaccine Components

An RSV soluble F (sF) protein containing amino acids 1–524 of the RSV A2 F sequence and lacking the transmembrane domain [23] was purified from the supernatants of stably transfected Chinese Hamster Ovary (CHO) cells [24]. RSV sF was quantified by Bradford assay and used both for immunizations and for ELISA assays. SDS-PAGE and western blot analysis indicated that RSV sF protein was >95% pure. Alum (aluminum hydroxide) adjuvant was obtained as Alhydrogel (Accurate Chemical and Scientific, NJ). Alum was used at 100 μg per vaccine dose, and adsorbed to protein by mixing for 30 minutes at 22°C. GLA in an aqueous formulation (GLA-AF, referred to as GLA), SE, and GLA-SE adjuvants were obtained from Immune Design Corporation (Seattle, WA) [25]. GLA in an aqueous formulation was used at 5 μg per vaccine dose. SE is a stabilized squalene-based emulsion with a mean particle size of ∼100 nm that was used at a 2% (weight/volume) concentration. Except where otherwise noted, GLA-SE was used at 5 μg GLA in 2% SE per dose. All vaccine formulations were prepared within 24 hours of inoculation.

RSV A2 strain (ATCC) was used for immunization and challenge. Virus was propagated in Vero cells grown with EMEM. Viral supernatants were centrifuged to remove cellular debris, stabilized with 1xSP (0.2 M sucrose, 0.0038 M KH_2_PO_4_, and 0.0072 M KH_2_PO_4_) and snap frozen in aliquots at −80°C until use. Virus titers were determined by plaque assay on Vero cell monolayers as previously described [26].

### Vaccination and Challenge

RSV-seronegative 7–10 week old female BALB/c mice or 5–6 week old female Sprague Dawley rats (Charles River Laboratories, Hollister, CA) and 6–8 week old female cotton rats (Harlan Laboratories, Indianapolis, IN) were housed under pathogen-free conditions at MedImmune. The MedImmune International Animal Care and Use Committee board approved this research, and all animal work was performed in accordance with the IACUC policies. Animals were lightly anesthetized with isoflurane for immunizations and blood draws and euthanized with carbon dioxide for terminal organ harvests.

Groups of mice were anesthetized and immunized twice, two weeks apart, with placebo (PBS) or RSV sF −/+ adjuvant in a 100 μl volume intramuscularly. Unless otherwise indicated, RSV sF was given at a dose of 0.3 μg, which had been determined from a titration study in mice to provide partial protection in the absence of adjuvant (S1 Fig.). Positive controls were inoculated intranasally with 10^6^ particle forming units (PFU) of RSV-A2 once at day 0. Mice were challenged with an intranasal administration of 10^6^ PFU of RSV A2 virus 14 days post the booster vaccination. Lungs were harvested 4 days post challenge unless otherwise noted. Cotton rat studies were similarly designed, except that the animals were immunized twice, three weeks apart, with RSV challenge at day 42. Sprague Dawley rat studies were similarly designed except that the animals were immunized twice, three weeks apart, in a 500 μl volume intramuscularly.

### Pulmonary RSV Quantitation by Plaque Titration

Viral titers in individual lung homogenates were quantified at 4 days post challenge by plaque assay. Fresh lungs excised from euthanized mice or cotton rats were weighed and homogenized in OptiMEM (Invitrogen) supplemented with 1xSP buffer using an OMNI tissue homogenizer with disposable heads (Omni International, Kennesaw, GA). Homogenates were clarified by centrifugation. Virus titers were determined by plaque assay on Vero cell monolayers as previously described [26]. Briefly, serial dilutions of freshly prepared lung homogenates were added to Vero cells in 6 well plates, allowed to infect for 1 hr, then overlaid with 1% methyl cellulose/EMEM and incubated for 5–7 days to allow plaque formation. Overlay was removed, cells were methanol-fixed, and plaques were visualized by staining with goat anti-RSV (Millipore, Billerica, MA), followed by HRP-rabbit anti-goat antibody and AEC (Dako, Glostrup, Denmark).

### RSV micro-neutralization assay

Sera were obtained from retro orbital blood collection, separated from whole blood and stored at −20°C until evaluated. RSV neutralizing antibody titers in heat-inactivated sera were measured using a GFP-tagged RSV A2 micro-neutralization assay as previously described [27]. Briefly, confluent Vero cell monolayers were infected with 500 PFU of virus alone or virus pre-mixed with serially diluted serum samples, then incubated at 33°C and 5% CO_2_ for 22 hrs. Plates were washed to remove unattached viruses and GFP fluorescent viral foci were enumerated using the IsoCyte image scanner (Blueshift, Sunnyvale, CA). Neutralizing titers are expressed as the log_2_ reciprocal of the serum dilution that resulted in a 50% reduction in the number of fluorescent foci (EC_50_ titers) as calculated using a 4-parameter curve fit algorithm.

### F-specific Serum ELISA

RSV F-specific IgG antibodies were assessed using standard ELISA techniques. High binding 96 well plates were coated with purified RSV sF. After blocking, serial dilutions of serum were added to plates. For mouse, bound antibodies were detected using HRP-conjugated goat anti-mouse IgG1 or IgG2a (Jackson ImmunoResearch, West Grove, PA) and developed with 3,3´,5,5´-tetramethylbenzidine (TMB, Sigma, St. Louis, MO). For rat, bound antibodies were detected using biotin-conjugated goat anti-rat IgG1, IgG2a, or IgG2b (Biolegend, San Diego, CA) followed by streptavidin-HRP (BD Biosciences, San Jose, CA) and developed with TMB. Absorbance was measured at 450 nm on a SpectraMax plate reader and analyzed using SoftMax Pro (Molecular Devices, Sunnyvale, CA). Titers are reported as log_2_ endpoint titers using a cutoff of 3x the mean of the blank wells.

### Cell isolation

Individual spleens were disrupted through a 100 micron nylon filter (Falcon) at the indicated harvest times. Viability of red blood cell depleted splenocytes was determined by ViCell and cells were resuspended at 10x10^6^ viable cells/mL in RPMI 1640 supplemented with 5% FCS, penicillin-streptomycin, 2 mM L-glutamine and 0.1% β-mercaptoethanol (cRPMI-5) prior to use.

Lung leukocytes were isolated from enzyme dispersed lung tissue at the indicated harvest times. Lungs were excised, washed in PBS, minced, and incubated for 45 minutes in RMPI 5% FCS, 1 mg/mL collagenase (Roche Applied Science) and 30 μg/mL DNase (Sigma, St Louis MO) prior to disruption through a 100 micron nylon filter (Falcon). Cells were washed and respuspended in cRPMI-5 and total viable cell counts were determined by ViCell.

### Cytokine profiling

For cytokine restimulation assays, splenocytes were incubated in 96 well plates with either medium alone (cRPMI-5) or with the pair of RSV F derived MHC II (I-E^d^)-binding peptides GWYTSVITIELSNIKE and VSVLTSKVLDLKNYI [9] (5 μg/mL each) for 72 hours. Supernatants were clarified by centrifugation and stored at-80°C until evaluated. Mouse cytokine/chemokine multiplex kits designed to include IFNγ, IL-5, IL-13, IL-17 and eotaxin (Millipore, Billerica, MA) were used to evaluate restimulated splenocyte supernatants and fresh lung homogenates. Lung homogenates were clarified by centrifugation prior to use. Assays were performed following manufacturer instructions and plates were analyzed on a Luminex reader (Bio-Rad, Hercules, CA). F-specific splenic cytokine production was determined by subtracting media alone values from F stimulated values.

### Histology sample preparation

Individual lung lobes were reserved and inflated with PBS + 4% paraformaldehyde for up to 1 week, then dehydrated and embedded in paraffin. Lung sections (5 microns) were prepared using a microtome from paraffin-embedded formalin-fixed lung lobes harvested from mice at 4 days post RSV challenge. Sections stained with hematoxylin and eosin were digitally scanned and examined by a licensed pathologist. Lung sections were evaluated for bronchiolar hyperplasia, alveolitis, and eosinophilic infiltrate.

### ELISPOT assays

Mabtech (Cincinnati, OH) pre-coated IFNγ ELISPOT kits were used for mouse and Sprague Dawley rat ELISPOT assays. For cotton rat IFNγ ELISPOT assays, 96 well PVDF plates (Millipore, Billerica, MA) were coated overnight with cotton rat anti-IFNγ capture antibody from R&D DuoSet ELISA Systems at 10 μg/mL in PBS. Coated microtiter plates were blocked with cRPMI-5, then 250,000 cells/well were incubated on blocked plates for 36–48 hours in triplicate with stimuli. For mouse ELISPOT assays stimuli used were media alone, MHC II (I-E^d^)-binding RSV F peptides GWYTSVITIELSNIKE and VSVLTSKVLDLKNYI [9] (5 μg/mL each), MHC I (H2-K^d^) binding RSV F peptide, KYKNAVTEL [9], and ConA (5 μg/mL) as a positive control. For cotton rat and Sprague Dawley rat ELISPOT assays the stimuli used were media, RSV sF (2 μg/mL), and ConA (5 μg/mL) as a positive control. Following incubation cells were washed away, plates were incubated with the appropriate biotinylated anti-IFNγ detection antibody followed by streptavidin-HRP, and spots were detected with TMB reagent (Mabtech kits) or with 3-amino-9-ethylcarbazole (AEC, Vector Labs, Burlingame, CA). Plates were read and analyzed using an ImmunoSpot reader and software (Cellular Technology Ltd, Cleveland, OH).

### Flow cytometry analysis

Red blood cell depleted splenocytes and lung leukocytes were distributed in 96 well microtiter plates at 10^6^ cells/well with media alone, MHC I (H2-K^d^) binding F peptide KYKNAVTEL (10 μg/mL), MHC I (H2-K^d^) binding M2 peptide SYIGSINNI (10 μg/mL), or ConA as a positive control. Cells were incubated at 37°C in 5% CO_2_ for 5–6 hrs, with Brefeldin A added an hour into the stimulation to block cytokine secretion. Cells were stained for viability with LIVE/DEAD violet, then with surface antibodies including CD3-PerCP-Cy5.5, CD8-PE-Cy7, and CD19-APC-Cy7. Following fixation with 2% paraformaldehyde and permeabilization with CellPerm (BD Bioscience, San Jose, CA), cells were stained with IFNγ-APC, IL-2 -FITC, and TNFα-PE. Cells were analyzed on a LSR 2 (BD Bioscience, San Jose, CA), collecting 10,000 CD8+ events.

### Statistics

Data was analyzed using Prism GraphPad software. Statistical significance was calculated either by one way ANOVA with Tukey’s multiple comparisons post test or by a Kruskal-Wallis test followed by a Dunn’s multiple comparisons post test, both with a cutoff of p<0.05.

## Results

### Adjuvanted RSV sF subunit vaccines confer protective immunity in BALB/c mice, with GLA-SE adjuvanted RSV sF inducing T_H_1-biased protective immunity

**Fig 1:**
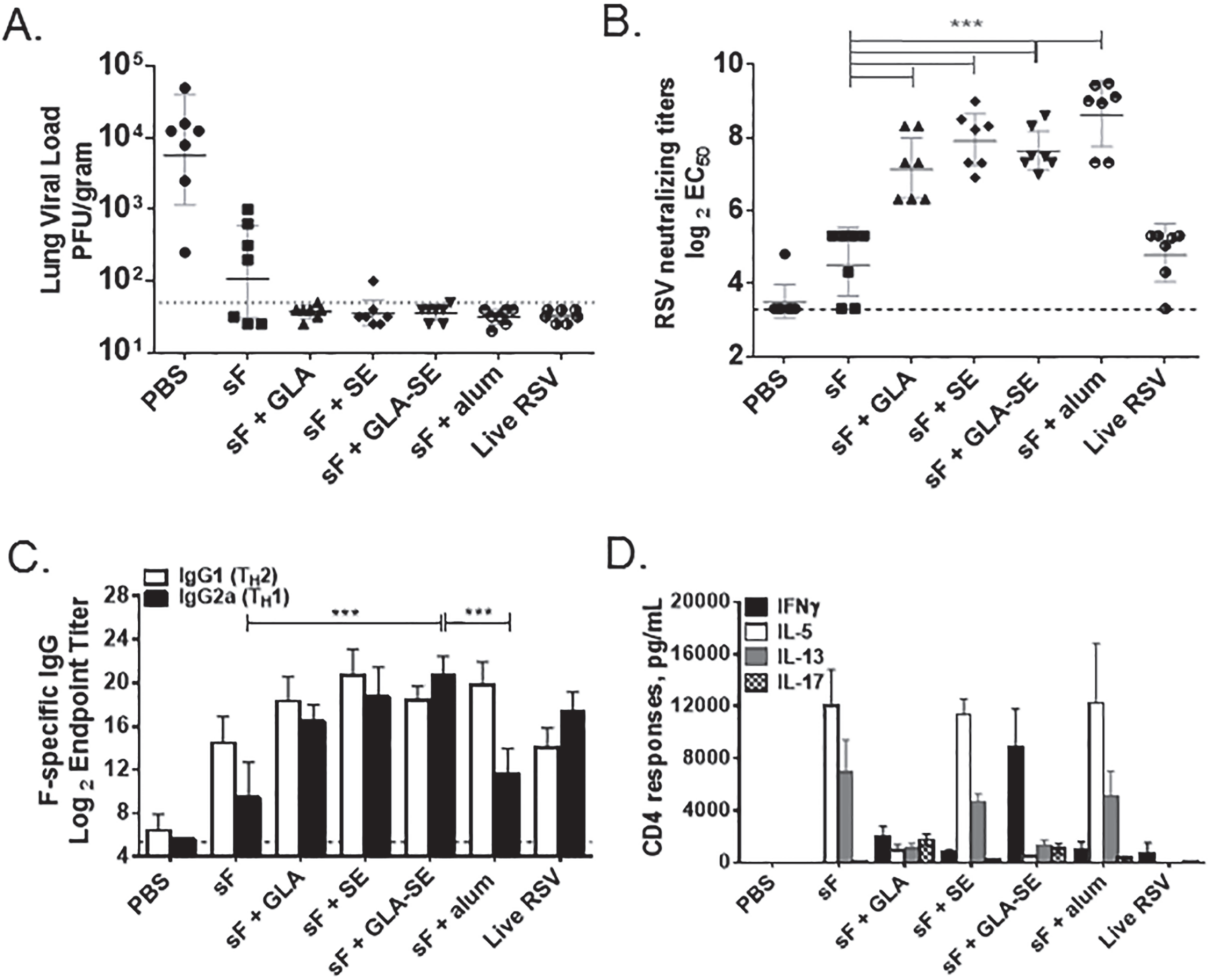
Adjuvanted RSV sF subunit vaccines induce protection, RSV neutralizing titers, and T_H_1 or T_H_2 responses in BALB/c mice. Mice (N = 7 per group) were immunized at days 0 and 14 with the indicated vaccines and challenged with 6 log_10_ PFU of RSV A2 at day 28. Representative data shown from 1 of 2 experiments. **(A) Lung Viral Titers**. Residual virus in the lungs of animals 4 days post challenge was quantified by plaque assay. Individual results are presented in log_10_PFU/mL, along with a bar representing the group geometric mean and a dotted line indicating the highest assay limit of detection (LOD), ∼1.4 log_10_ PFU/mL. Individuals with undetectable titers were scored at the LOD. **(B) Serum RSV Neutralizing Titers**. Individual Day 28 sera results are presented as the log_2_ dilution of serum that provides 50% reduced fluorescent focus units (FFU) of RSV-GFP infection, with a bar representing the group geometric mean. Individuals with undetectable titers were scored at the assay LOD of 3.3 log_2_, indicated by a dashed line. Significant differences (by 1 way ANOVA) are indicated by ***. **(C) Serum F-specific IgG1 and IgG2a Titers**. Day 28 sera (n = 7 per group) were evaluated for F-specific IgG1 and IgG2a isotypes by endpoint titer ELISA. Data is presented as the log_2_ reciprocal serum endpoint dilution with a LOD of 5.64. Shown is the group geometric mean with 95% confidence interval, with significant differences between groups (by 1 way ANOVA) indicated by ***. **(D) F-specific CD4 T-cell Cytokine Responses**. Splenocytes (n = 3 per group) were harvested 4 days post challenge and restimulated 72 hours with a pair of immunodominant MHC II restricted RSV F peptides. Shown are the F-specific IFNγ, IL-5, IL-13, and IL-17 responses calculated by subtracting media control values from test values in multiplexed cytokine analysis. The group means and SEM are shown.

Adjuvanted RSV sF vaccines were first evaluated in the BALB/c mouse model for their ability to induce RSV A2 protective immunity, measured by a >100-fold reduction in lung viral titers 4 days post challenge in comparison to a PBS negative control. BALB/c mice were immunized intramuscularly with two doses of RSV sF subunit vaccines without adjuvant or adjuvanted with alum, GLA, SE, or GLA-SE. Positive controls were primed with one dose of live RSV intranasally. Following challenge, all vaccines provided significant lung viral titer decreases compared to PBS, which had a mean lung viral titer of 3.8 log_10_ PFU/gram (Fig. 1A). Immunization with 0.3 μg unadjuvanted RSV sF provided partial lung protection to mice, with 4 of 7 animals having detectable lung viral titers ranging from 2.3–3.0 log_10_ PFU/gram, while the adjuvanted RSV sF vaccines provided complete lung protection, with mean viral titers below the limit of detection of the plaque assay.

Serum RSV neutralizing titers were significantly enhanced with all adjuvanted RSV sF vaccines. While both unadjuvanted RSV sF and intranasal infection with live RSV virus induced detectable serum neutralizing titers (geometric means of 4.5 log_2_ and 4.8 log_2_ respectively), these responses were not significantly different from the PBS negative control group. In contrast, the four adjuvanted RSV sF groups induced geometric mean neutralizing titers > 4-fold higher than achieved with RSV infection and significantly greater than observed in the PBS group, at 7.1 log_2_ for sF + GLA, 7.9 log_2_ for sF + SE, 7.6 log_2_ for sF + GLA-SE, and 8.6 log_2_ for sF + alum (Fig. 1B). ELISA titers for total serum F-specific IgG showed a similar trend (S2 Fig.).

To evaluate T_H_1/T_H_2 type responses to these vaccine formulations, we measured the isotypes of F-specific antibodies from each animal. IFNγ promotes class-switching of antibodies from IgG1 to IgG2a in the mouse, making IgG2a antibodies a good correlate of T_H_1-biasing IFNγ. IgG2a antibody titers were significantly higher with sF + GLA-SE compared to sF or sF + alum (Fig. 1C). F-specific IgG2a titers were greater than F-specific IgG1 titers for only two groups, sF + GLA-SE and live RSV positive control.

Next, the cytokine profile of supernatants from restimulated splenocytes was assessed to better evaluate systemic T_H_1/T_H_2/T_H_17 type responses to these vaccine formulations. F-specific CD4+ T cells were restimulated using MHC II (I-E^d^)-binding RSV F derived peptides. IFNγ was evaluated as the prototypical T_H_1-type cytokine, IL-5 and IL-13 as representative T_H_2-type cytokines and IL-17 as a T_H_17-type cytokine. Splenocytes from PBS control animals demonstrated no F- specific cytokine production, while those from live RSV infected mice demonstrated a weak IFNγ response (Fig. 1D). The sF + GLA-SE vaccine group induced an RSV F-specific response dominated by IFNγ indicative of a T_H_1-type response and consistent with the observed enhancement in the IgG2a antibody response. In contrast, the sF + GLA group demonstrated a balanced F-specific response that included T_H_1, T_H_2, and T_H_17 cytokines, while sF, sF + SE, and sF + alum groups demonstrated a T_H_2-type response characterized by IL-5 and IL-13 cytokines.

### GLA-SE adjuvanted RSV sF vaccines promote lung T_H_1 responses following RSV challenge in BALB/c mice

**Fig 2:**
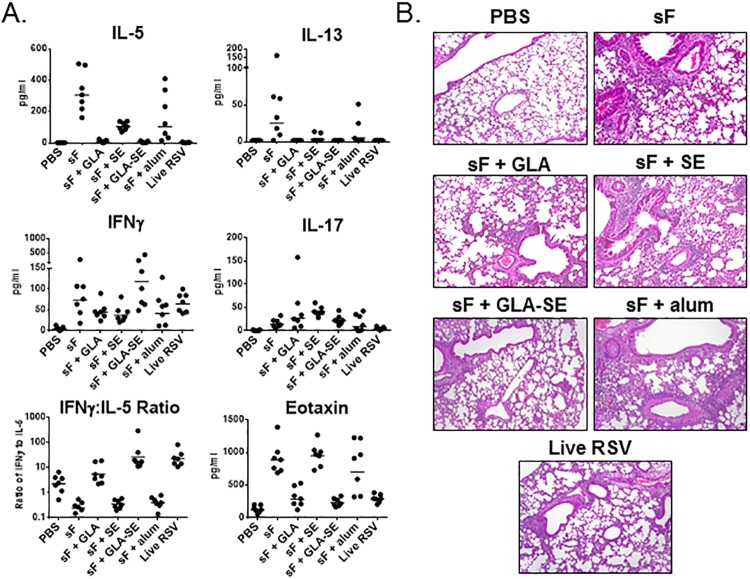
Systemic T_H_1 or T_H_2 responses induced by adjuvanted RSV sF subunit vaccines are reflected in lung responses to RSV challenge in BALB/c mice. Mice (n = 7 per group) were immunized at days 0 and 14 with the indicated vaccines and challenged with 6 log_10_ PFU of RSV A2 at day 28. Lungs were harvested 4 days post challenge. Representative data shown from 1 of 2 experiments run with all groups. **(A) Cytokines in Lung Homogenates**. Levels of IL-5, IL-13, IFNγ, IL-17, and eotaxin in clarified lung homogenates were quantified by multiplexed cytokine analysis and calculated as the amount per gram of lung harvested. Individual mouse results are shown, along with a bar representing the group mean. To calculate the IFNγ to IL-5 ratio, values were first zero-adjusted by adding 1 to each value before calculating. **(B) Pulmonary Cellular Infiltration**. Formalin-fixed lung sections were H&E stained and evaluated for eosinophilic inflammation. Shown are representative 10x field views from each group.

To further assess the cytokine profile in the local lung environment following viral challenge, we measured IL-5, IL-13, IFNγ, IL-17, and eotaxin in individual lung homogenates harvested 4 days post RSV challenge. These cytokine readouts provide a snapshot of the cytokines made by any lung-recruited immune cells, including macrophage, eosinophils, B cells, and T cells. PBS-immunized animals had low levels of all tested cytokines. IL-5 and IL-13 were only detected in the lungs of mice immunized with unadjuvanted sF, sF + SE, or sF + alum, while IFNγ was detected in the lungs of all mice except those in the PBS group (Fig. 2A). The average ratio of IFNγ to IL-5 can express the T_H_1/T_H_2 response character with a ratio > 1.0 indicating a more T_H_1-type response. T_H_1-type responses were observed with sF + GLA (ratio: 7.8), sF + GLA-SE (ratio: 59.3), and live RSV (ratio: 29.2). A T_H_2-type response was observed for unadjuvanted sF (ratio: 0.3), sF + SE (ratio 0.4), and sF + alum (ratio: 0.5) (Fig. 2A). Only low levels of IL-17 were detected in immunized mice, and these did not vary significantly with the use of adjuvants. These results indicate that sF + GLA-SE induces a lung-localized T_H_1 response as well as a systemic T_H_1-type immune response, while sF alone, sF + SE, and sF + alum induce more of a T_H_2–type response in the lung.

T_H_2-type responses to RSV challenge in the BALB/c lung, particularly those characterized by IL-13 production, can correlate with increased eotaxin (CCL11) and eosinophilic infiltration in the lungs [28, 29]. In this study, pulmonary eotaxin was at <300 pg/mL in the PBS and live RSV groups (Fig. 2A). Elevated pulmonary eotaxin (mean >750 pg/mL) was observed in groups with T_H_2-type immune responses (sF alone, sF + SE, and sF + alum). In contrast, pulmonary eotaxin levels in groups with T_H_1-type immune responses (sF + GLA and sF + GLA-SE) were at baseline (mean <300 pg/mL). Examination of lung sections revealed minimal eosinophilic infiltration in the lungs of PBS animals (experiencing a primary infection with RSV) or live RSV animals (experiencing a secondary RSV infection) (Fig. 2B). Animals that received GLA-SE adjuvanted RSV sF formulations also had minimal eosinophilic infiltration, similar to the mice primed with RSV infection. However, animals vaccinated with unadjuvanted sF, sF + SE, or sF + alum had increased eosinophilic infiltration and other pulmonary lesions. These data demonstrate that the systemic T_H_1-biased immune response achieved by immunization with sF + GLA-SE corresponds with a lung T_H_1-biased immune response characterized by low lung eotaxin levels, and limited pulmonary eosinophilia following RSV challenge in naive BALB/c mice.

### GLA-SE adjuvanted RSV sF vaccines prime robust CD8 T cell responses following viral challenge

**Fig 3:**
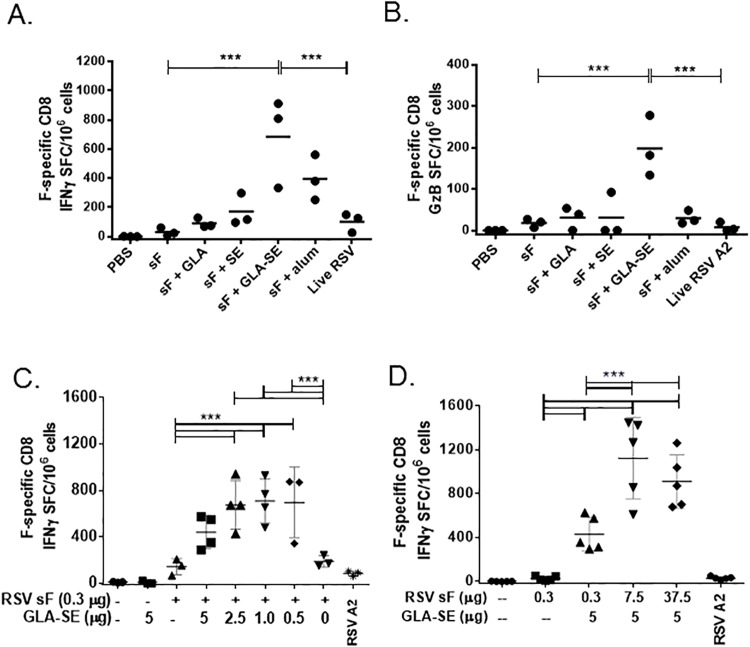
GLA-SE adjuvanted RSV sF vaccines prime F-specific CD8 responses in BALB/c mice. Mice (N = 3–5 per group) were immunized at days 0 and 14 with the indicated vaccines and challenged with 6 log_10_ PFU of RSV at day 28. Splenocytes were harvested at 4 days post challenge and restimulated with an immunodominant RSV F-derived MHC I restricted peptide in ELISPOT. (**A) Effect of different adjuvants on IFNγ ELISPOT**. Individual mouse results are shown, along with a bar representing the group mean, for 3 animals/group immunized with 0.3μg RSV sF formulations in a representative experiment (each group repeated 2–7 times). Significant differences between groups (by 1 way ANOVA) are indicated by ***. (**B) Effect of different adjuvants on Granzyme B ELISPOT**. Individual mouse results are shown, along with a bar representing the group mean, for 3 animals/group immunized with 0.3 μg RSV sF formulations. Significant differences between groups (by 1 way ANOVA) are indicated by ***. **(C) Effect of GLA-SE adjuvant dose titration on IFNγ ELISPOT**. Individual mouse results are shown, along with a bar representing the group mean, for 3–4 animals/group given a fixed 0.3 μg amount of RSV sF with the indicated doses of GLA in 2% SE. Absence of adjuvant is indicated by a dashed line, while SE given with no GLA is indicated by a 0. Significant differences between groups (by 1 way ANOVA) are indicated by ***. **(D) Effect of RSV sF antigen dose titration on IFNγ ELISPOT**. Individual mouse results are shown, along with a bar representing the group mean, for 3–4 animals/group given indicated doses of RSV sF formulated with or without a fixed amount of GLA-SE (5 μg GLA in 2% SE). Significant differences between groups (by 1 way ANOVA) are indicated by ***.

T_H_1-type vaccine responses observed with sF + GLA-SE may support the development of strong CD8 T cell responses. IFNγ ELISPOT with an immunodominant MHC I (H2-K^d^) binding F-derived peptide [9] was used to evaluate splenic CD8 T-cell responses in representative animals from each vaccine group following RSV challenge. PBS and unadjuvanted RSV sF groups had low (<30) F-specific CD8 IFNγ ELISPOT spot forming counts (SFC)/million cells (Fig. 3A). In contrast, sF + GLA-SE induced significantly greater F-specific CD8 IFNγ ELISPOT responses (mean: 684 SFC/million, a 23-fold increase relative to unadjuvanted sF). Other adjuvanted sF vaccine formulations did not increase CD8 responses significantly compared to unadjuvanted sF. Live RSV infection generated a weak F-specific CD8 response of only 100 SFC/million. This is not unexpected as the immunodominant RSV A2 response in the BALB/c mouse is against an M2-derived peptide [9]. In addition, F-specific Granzyme B secretion by ELISPOT was evaluated as a potential CD8 cytotoxicity readout. Only mice that received sF + GLA-SE demonstrated F-specific Granzyme B responses (mean 197 SFC/million) significantly above those of mice given unadjuvanted sF (mean 18 SFC/million) (Fig. 3B). Since polyfunctional T cells that co-express IFNγ TNFα (an effector cytokine) and IL-2 (a cytokine associated with proliferation) are reported to be the most effective at viral clearance, followed by T cells that co-express both IFNγ and TNFα [30], we also evaluated F-specific CD8 T cells by intracellular cytokine staining. The RSV sF + GLA-SE vaccine group had the highest numbers of both triple positive and IFNγ TNFα double positive splenic CD8 T cells (S3 Fig.).

Additional studies in BALB/c mice demonstrated that CD8 T cell responses following sF + GLA-SE vaccination are observed over a range of antigen and adjuvant doses. Mice that received 0.3 μg RSV sF with an adjuvant dose of 1–2.5 μg GLA-SE demonstrate significantly enhanced numbers of splenic F-specific CD8 T cells at 4 days post challenge compared to mice that received unadjuvanted sF or sF + SE alone (Fig. 3C). Animals that received 0.3, 7.5, or 37.5 μg sF + GLA-SE (5 μg/2%) also generated strong splenic F-specific CD8 T cells compared to PBS controls at 4 days post challenge (Fig. 3D). These dose-ranging studies suggested that for mouse CD8 T cell responses, an optimal RSV sF dose may be ≥ 7.5 μg and an optimal adjuvant dose may be 1–2.5 μg GLA-SE.

### GLA-SE adjuvanted RSV sF vaccines induce F-specific CD4 and CD8 T cell responses without viral exposure

**Fig 4:**
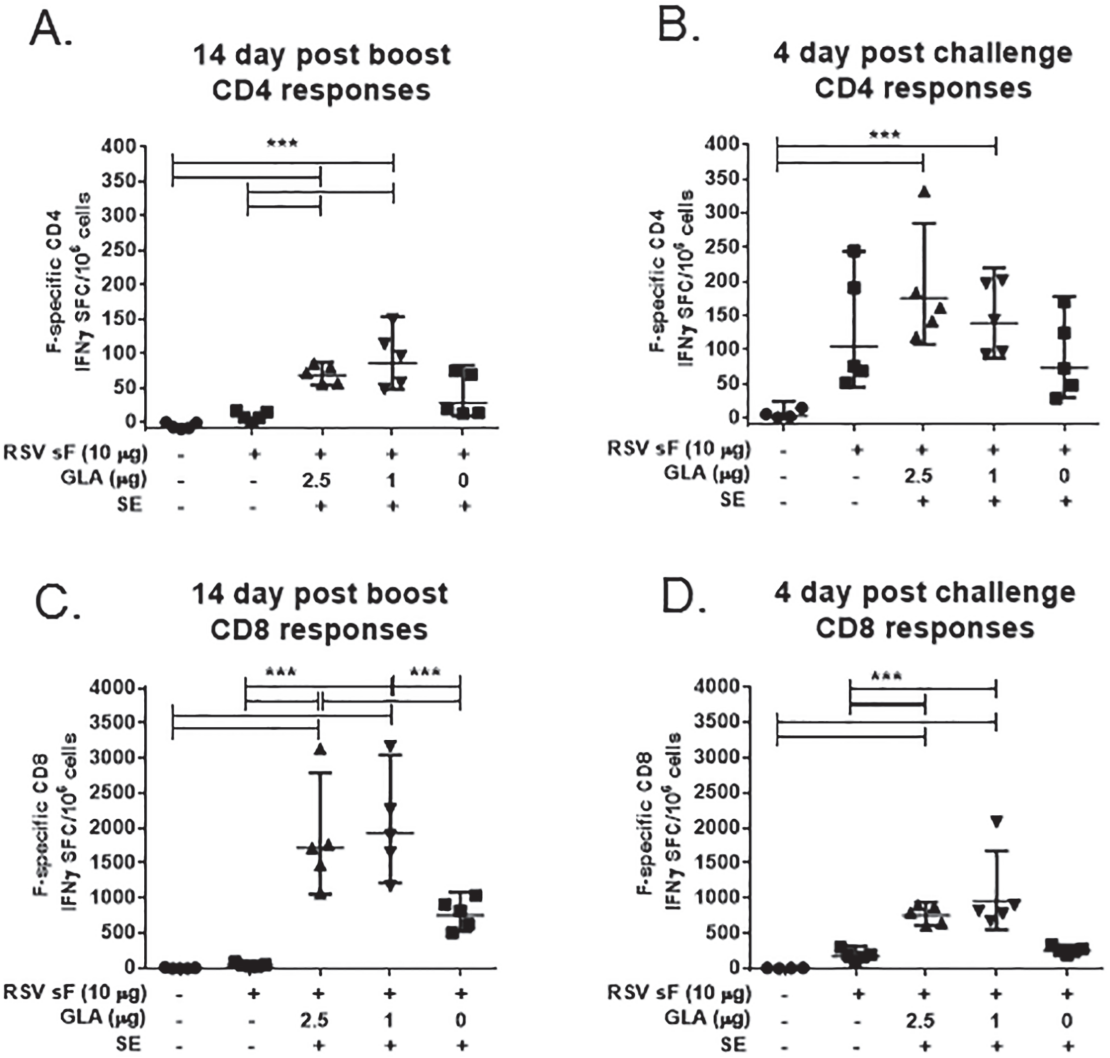
GLA-SE adjuvanted RSV sF vaccines induce F-specific CD4 and CD8 T cells without RSV challenge. Mice were immunized intramuscularly at days 0 and 14 with 10 μg of RSV sF alone or formulated with the indicated GLA-SE or SE adjuvants. Splenocytes (n = 5 per group) were harvested and restimulated with either a pair of MHC II-restricted F peptides (for CD4 responses, **A**&**B**) or an immunodominant MHC I-restricted F peptide (for CD8 responses, **C**&**D**). For **(A)** and **(C)**, splenocytes were harvested at day 28 (14 days post boost). For **(B)** and **(D)**, animals were challenged at day 28 with 6 log_10_ RSV A2 and splenocytes were harvested at day 32, 4 days post challenge.

In mice, post challenge F-specific T cells primed by sF + GLA-SE vaccines were detected over a range of RSV sF doses, as shown in Fig. 3. To evaluate T cell induction in the absence of RSV challenge, optimized doses of RSV sF (10 μg) and GLA-SE (1–2.5 μg in 2% SE) were used. Mice were vaccinated with sF, sF + GLA-SE or sF + SE at day 0 and day 14, with cohorts evaluated both at 14 days post second vaccine dose (boost) and at 4 days post RSV challenge. At 14 days post boost, both sF + GLA-SE groups had significantly stronger F-specific CD4 (mean 49–56 SFC/million) and CD8 (mean 1832–2033 SFC/million) T cell IFNγ responses compared to the PBS or unadjuvanted sF groups (Fig. 4, A and C). Post RSV challenge, F-specific CD4 IFNγ responses in sF + GLA-SE vaccinated animals were still significantly stronger than those seen in the PBS groups, and F-specific CD8 T cell IFNγ responses were still significantly stronger than in either the PBS or the unadjuvanted sF groups (Fig. 4, B and D). Together, these data indicate that immunization with RSV sF protein adjuvanted with GLA-SE elicits a systemic F-specific CD4 and CD8 T-cell response prior to live RSV exposure. Interestingly, the absolute numbers of F-specific splenic CD8 appeared lower in the post challenge cohort compared to the pre-challenge cohort, potentially indicating a relocalization of these CD8 cells to the site of viral challenge.

### CD8 T cell responses induced by vaccination with GLA-SE adjuvanted RSV sF vaccines are recruited to the lungs following RSV challenge

**Fig 5:**
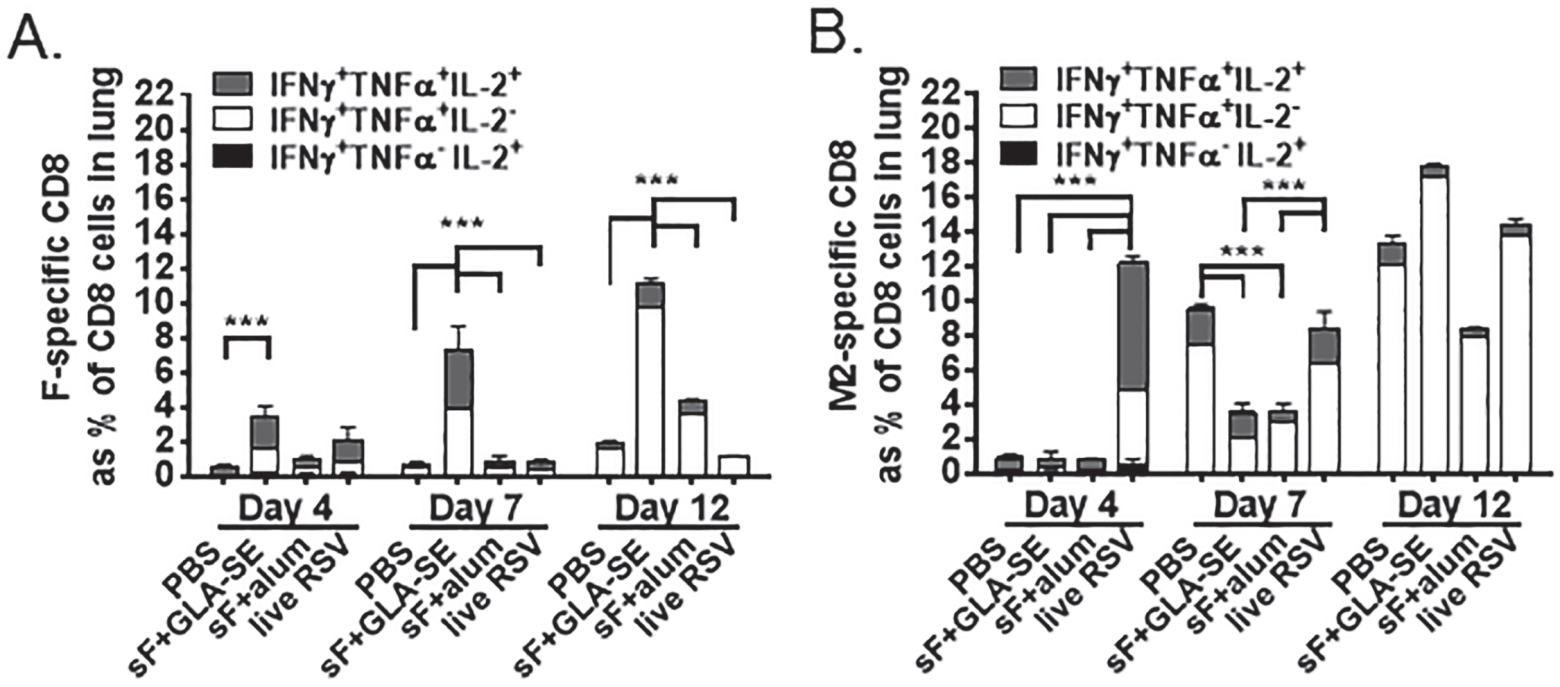
RSV sF + GLA-SE induces F-specific CD8 T cells that traffic to the lung following RSV challenge. Mice were immunized with the indicated RSV sF (0.3 μg) vaccine formulations at days 0 and 14 or with live RSV at day 0 and challenged with 6 log_10_ PFU of RSV at day 28. Lungs were harvested 4, 7, or 12 days post challenge (n = 3 for each group and timepoint) and restimulated 6 hours with either **(A)** an RSV F-derived H-2K^d^ restricted peptide or **(B)** an RSV M2-derived H-2K^d^ restricted peptide as a positive control. Cells were surface stained for CD3 and CD8, intracellularly stained for IFNγ, TNFα, and IL-2, and analyzed on an LSR2 for the frequency of responding CD8 T cells. The group mean is shown with significant differences between groups (by 1 way ANOVA) indicated by ***. Representative data from 1 of 2 experiments is presented.

To characterize the potential relocalization of vaccine-induced CD8 T cells to the site of viral replication, lung leukocytes at days 4, 7 or 12 post RSV A2 challenge were harvested for flow cytometric analysis from mice vaccinated with PBS, sF + GLA-SE, sF + alum, or live RSV. CD3+ T cells were the major viable leukocyte population observed in each cohort and were evaluated for functional cytokine responses to antigen restimulation. By 4 days post challenge, mice vaccinated with sF + GLA-SE already had significantly greater F-specific functional CD8 T cells in the lungs compared to mice that received PBS (3.4% vs 0.5%) (Fig. 5A). By 7 days post challenge, mice vaccinated with sF + GLA-SE had 7.3% F-specific functional CD8 T cells in the lungs, a significant difference from the 0.4% F-specific CD8 T cells observed in mice administered PBS, the 0.9% observed in mice vaccinated with sF + alum, or the 0.8% observed in mice previously dosed with live RSV (Fig. 5A). At 4 and 7 days post challenge, F-specific functional CD8 T cells were equally split between triple positive for IFNγ, TNFα, and IL-2 and double positive for IFNγ and TNFα. At 12 days post challenge, the frequency of lung-localized F-specific functional CD8 T cells in the sF + GLA-SE group was still significantly greater than observed in other groups, although the predominant T cell population had lost IL-2 production and was only double positive for IFNγ and TNFα. T cells that lack IL-2 are less proliferative, so these cells could represent one of the first steps of the contraction phase. These data indicate enhanced recruitment of polyfunctional F-specific T cells to the lung following RSV challenge in the sF + GLA-SE group compared to either control PBS immunized animals, sF +alum immunized animals, or even live RSV infected animals.

As a control, immunodominant M2-specific functional CD8 responses were evaluated in parallel in these animals. M2-specific responses in the lung developed rapidly in mice previously infected with live RSV, comprising 12% of the total lung CD8 population by 4 days following RSV reinfection. At day 4, these CD8 cells were primarily triple positive for IFNγ, TNFα, and IL-2 (Fig. 5B). At later timepoints the number of M2-specific functional CD8 T cells in the live RSV immunized group did not change significantly, but IFNγ/TNFα double positive CD8 T cells became predominant. By days 7–12 following RSV challenge the number of M2-specific CD8 T cells had increased in the PBS, sF + GLA-SE and sF + alum immunized groups, indicating an induction of CD8 T cells to this immunodominant epitope in BALB/c mice upon intranasal exposure to RSV. Delayed kinetics of M2-specific CD8 T cell responses in the sF + GLA-SE and sF + alum groups versus the PBS group may reflect the contribution of F-specific antibodies and cellular responses to the control of viral replication.

### RSV sF + GLA-SE completely protects naive cotton rats from RSV challenge and induces both RSV neutralizing titers and F-specific cellular IFNγ responses

The cotton rat is another well-established RSV challenge model, more permissive to RSV replication than mice, although with fewer immunological reagents for characterization of immune responses. To confirm the immunological profile of GLA-SE adjuvanted RSV sF in a second RSV challenge model, individual cotton rats were administered RSV sF subunit vaccines without adjuvant or adjuvanted with GLA, SE, GLA-SE, or alum. One group of cotton rats was immunized with GLA-SE alone as a negative control, while another group was given one intranasal dose of 1 x 10^6^ PFU of live RSV A2 virus at day 0 as a positive control.

**Fig 6:**
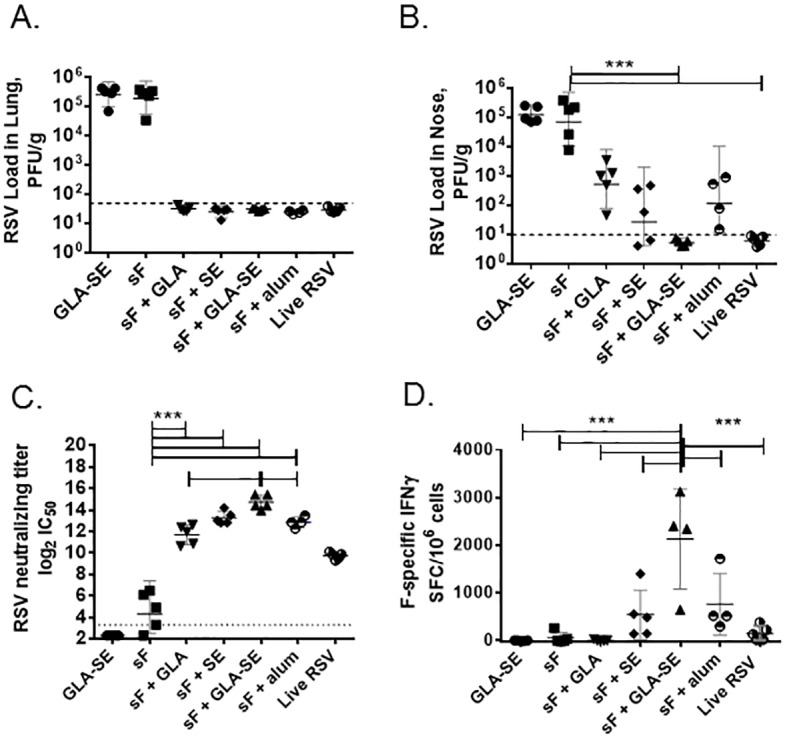
Adjuvanted RSV sF subunit vaccines induce protection, RSV neutralizing titers, and F-specific IFNγ responses in cotton rats. Animals were immunized at day 0 and day 21 with the indicated vaccine formulations (using 0.3 μg of RSV sF per immunization) and challenged at day 42 with 6 log_10_ PFU of RSV. **(A) Lung Viral Titers**. Lungs were harvested at 4 days post challenge from individual animals (n = 8 per group) with residual virus quantified by plaque forming assay. Individual results are shown in log_10_, along with a bar representing the group mean. The dotted line indicates the highest assay LOD. Significant differences (by 1 way ANOVA) between individual groups and the Live RSV A2 group are indicated by ***. **(B) Nose Viral Titers**. Noses and nasal turbinates were harvested at 4 days post challenge from individual animals (N = 8 per group) with residual virus quantified by plaque forming assay. Individual results are shown in log_10_, along with a bar representing the group mean. The dotted line indicates the highest assay LOD. Significant differences (by 1 way ANOVA) between individual groups and the Live RSV A2 group are indicated by ***. **(C) Serum RSV Neutralizing Titers**. Day 42 sera (N = 5 per group) were heat inactivated and tested for neutralization of RSV-GFP infection of target cells by fluorescent focus assay. Data is presented as the log_2_ dilution of serum that provides 50% reduced fluorescent focus units (FFU) of virus with a LOD of 3.3 indicated by a dashed line. Individual results are shown, along with a bar representing the group mean and 95% confidence interval. Significant differences (by 1 way ANOVA) between individual vaccine groups are indicated by ***. **(D) IFNγ ELISPOT**. Splenocytes (N = 4–5 per group) harvested 4 days post challenge were restimulated with either media or with RSV sF protein in an IFNγ ELISPOT. F-specific responses were quantified by subtracting the media control values from the test values. Significant differences (by 1 way ANOVA) between individual vaccine groups are indicated by ***.

In contrast to what was observed in mice, immunization with a 0.3 μg dose of unadjuvanted RSV sF did not provide RSV protection in cotton rat lungs at 4 days post RSV A2 challenge. Negative control animals demonstrated a viral load of 5.5 log_10_ PFU/gram, while all adjuvanted RSV sF vaccines demonstrated complete lung protection equivalent to live RSV (Fig. 6A). Interestingly, adjuvanted RSV sF vaccines also demonstrated protection of the upper respiratory tract in cotton rats. Whereas negative control animals had a high viral titer of 5.1 log_10_ PFU/gram in the nose, sF + GLA-SE conferred complete protection in the nose equivalent to live RSV (Fig. 6B). Partial protection of the upper respiratory tract was observed in groups that received sF + GLA (mean 2.7 log_10_ PFU/gram), sF + SE (mean 1.4 log_10_ PFU/gram), or sF + alum (mean 2.1 log_10_ PFU/gram). These data further confirmed the superiority of sF + GLA-SE over other adjuvanted sF vaccines at the primary site of RSV infection.

RSV neutralizing titers were induced by all adjuvanted RSV sF formulations at levels greater than those achieved by either unadjuvanted RSV sF (4.3 log_2_) or an intranasal infection with live RSV A2 virus (9.7 log_2_)(Fig. 6C). Of note, sF + GLA-SE induced the highest neutralizing titers of all the tested formulations, with a geometric mean of 14.7 log_2_. These titers were significantly higher than the titers achieved with sF + alum (12.9 log_2_) or sF + GLA (11.7 log_2_), though not different from those observed for the sF + SE vaccine group (13.3 log_2_).

Next, F-specific cellular responses were measured in the cotton rat by IFNγ ELISPOT. sF + GLA-SE induced the strongest F-specific IFNγ response (mean: 2626 SFC/million cells), a 45-fold increase over unadjuvanted RSV sF (mean: 58 SFC/million) and a significantly stronger response than seen with any other tested formulation (Fig. 6D). RSV F-specific IFNγ responses observed with sF + GLA (mean: ∼7 SFC/million), sF + SE (mean: ∼642 SFC/million) or sF + alum (mean: 1246 SFC/million) were not significantly above those of the unadjuvanted RSV sF group. In contrast, live RSV infection generated a splenic F-specific IFNγ ELISPOT response of only 196 SFC/million. Concomitant measurements of splenic F-specific IL-4 responses (S4 Fig.) indicated that sF + GLA-SE generated the most T_H_1-biased response of all the tested formulations including live RSV infection. In combination, these results in cotton rats further demonstrate that sF + GLA-SE induces robust neutralizing antibodies and T_H_1-biased cellular responses that are conducive to viral clearance in the lung.

### RSV sF + GLA-SE induces RSV neutralizing titers, a T_H_1-bias, and F-specific cellular IFNγ responses in Sprague Dawley rats

Sprague Dawley rats are often used in toxicology studies for drug and vaccine development, as their body weight allows administration of human clinical doses. Sprague Dawley rats have been reported to be permissive for RSV infection [31]. We confirmed that intranasal inoculation with RSV A2 resulted in lung viral titers in Sprague Dawley rats (S5 Fig.). In order to characterize immune responses at the anticipated clinical doses, individual rats were primed and boosted with 10 or 100 μg of RSV sF with or without GLA-SE (2.5 μg GLA in 2% SE). Negative control groups of rats were administered PBS or GLA-SE alone, while a positive control group was given one intranasal dose of 1 x 10^6^ PFU of live RSV A2 virus at day 0.

**Fig 7:**
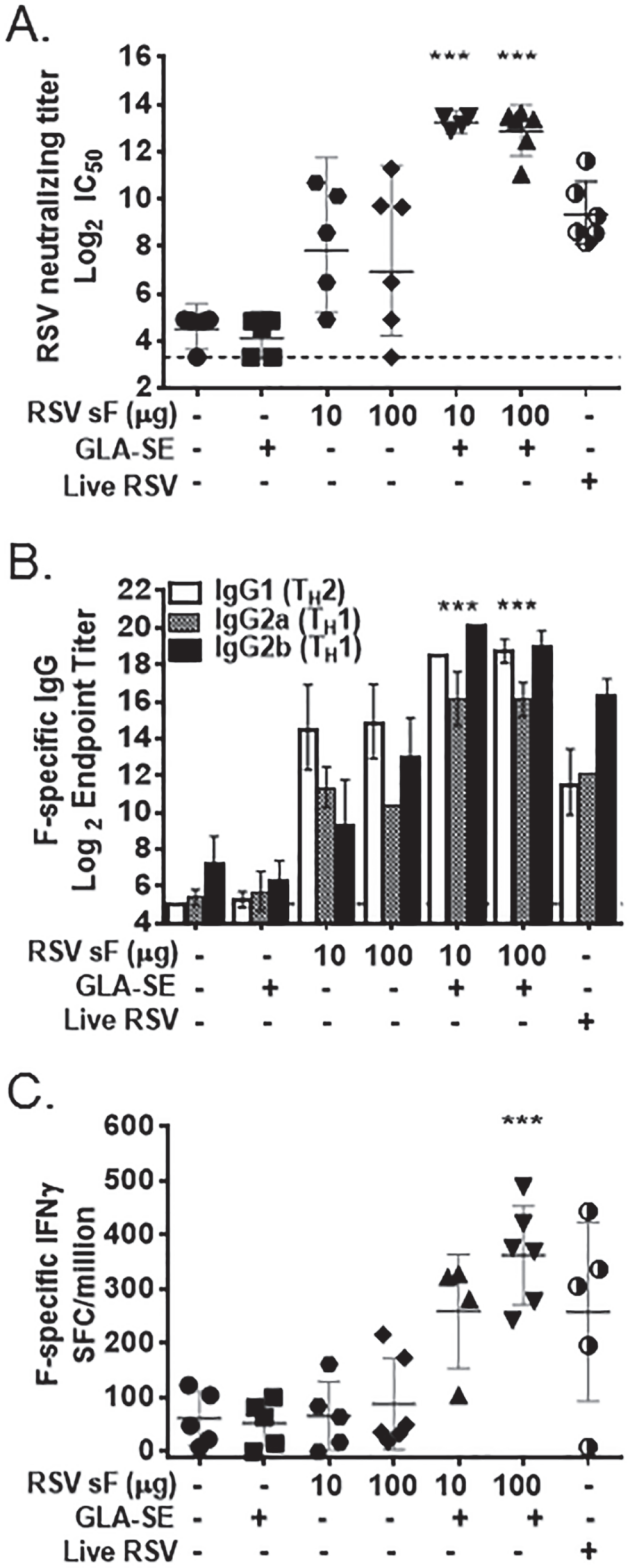
RSV sF + GLA-SE induces RSV neutralizing titers, a T_H_1-bias, and F-specific IFNγ responses in Sprague Dawley rats. Sprague Dawley rats were immunized at days 0 and 21 with the indicated vaccines and evaluated at day 42. Significant differences (by 1 way ANOVA) between RSV sF + GLA-SE vaccine groups and paired unadjuvanted RSV sF groups are indicated by ***. **(A) Serum RSV Neutralizing Titers**. Day 42 sera (N = 5 per group) were heat inactivated and tested for neutralization of RSV-GFP infection of target cells by fluorescent focus assay. Data is presented as the log_2_ dilution of serum that provides 50% reduced fluorescent focus units (FFU) of virus with a LOD of 3.3 indicated by a dashed line. Individual results are shown, along with a bar representing the group mean and 95% confidence interval. **(B) Serum F-specific IgG1, IgG2a and IgG2b Titers**. Day 42 sera (n = 5 per group) were evaluated for F-specific IgG1, IgG2a, and IgG2b isotypes by endpoint titer ELISA with a LOD of 5.64. Data is presented as the group geometric mean of the log_2_ reciprocal serum endpoint dilution with 95% confidence interval. **(C) IFNγ ELISPOT**. Splenocytes (N = 4–5 per group) were restimulated with either media or with RSV sF protein in an IFNγ ELISPOT. F-specific responses were quantified by subtracting the media control values from the test values.

As expected, sF + GLA-SE induced strong RSV neutralizing titers in rats, with a geometric mean of 12.9–13.3 log_2_ after the second dose (Fig. 7A). These titers were significantly higher than the titers achieved with unadjuvanted RSV sF (mean 7.0–7.8 log_2_), PBS (mean 4.5 log_2_), GLA-SE alone (mean 4.1log_2_) or intranasal infection with live RSV A2 virus (mean 9.3 log_2_), indicating the superiority of sF + GLA-SE at inducing high titer serum RSV neutralizing antibodies.

Sprague Dawley rats, like mice and cotton rats, also mounted a T_H_1-biased immune response. F-specific IgG2a and IgG2b titers (T_H_1-type subtypes) were significantly higher in rats that received RSV sF + GLA-SE vaccines compared to those that received unadjuvanted RSV sF (Fig. 7B). Overall, the enhancement in IgG2a titers (∼30-fold) and IgG2b titers (>100-fold) was greater than the increase in IgG1 titers (∼15-fold), further confirming that GLA-SE promotes a T_H_1-bias immune response in rodents. RSV sF + GLA-SE also induced the strongest F-specific IFNγ ELISPOT response (mean: 259–363 SFC/million cells), a >4-fold increase over unadjuvanted RSV sF (mean: 48–61 SFC/million) (Fig. 7C). These results provide further confirmation that RSV sF + GLA-SE induces high RSV neutralizing antibodies and F-specific IFNγ responses in naïve rodents.

## Discussion

This study explores the immunogenicity of vaccine formulations consisting of subunit RSV sF adjuvanted with GLA, SE, GLA-SE or alum as potential RSV vaccine candidates. We demonstrate that intramuscularly administered vaccines consisting of purified sF protein + GLA-SE are highly immunogenic, generating both high RSV neutralizing titers and a robust T_H_1-biased cellular response in BALB/c mice, cotton rats and Sprague Dawley rats, while completely protecting BALB/c mice and cotton rat from RSV challenge. In contrast, sF + alum or sF + SE induced a protective response characterized by RSV neutralizing titers but a weak and T_H_2-biased cellular response.

GLA-SE incorporates the TLR4 agonist GLA with the oil-in-water emulsion SE. This incorporation of a particulate delivery system with a TLR agonist may be ideal at inducing both strong T_H_1-biased humoral and cellular responses to associated subunit protein antigens. Previously, induction of strong CD8 T cell responses has been observed using enriched RSV proteins adjuvanted with an LPS-based particulate adjuvant [20]. Particulate emulsions are hypothesized to promote uptake of vaccine antigens by antigen presenting cells, while TLR4 triggering promotes both antibody production and T cell immunity [32–34]. Cytokines induced by TLR4 signaling, such as IL-6 and IFNγ, act as B cell growth factors and support class-switching to antibodies optimized for interactions with Fc receptors and complement [35, 36]. These and other TLR4-induced cytokines recruit professional antigen presenting cells, inducing up-regulation of MHC I molecules and antigen processing proteins resulting in better activation of T cells [37, 38]. Type I IFN induced by TLR4 signaling can also enhance cross-presentation of protein antigens [39], allowing induction of strong CD8 T cell responses to associated proteins [40, 41]. GLA-SE, like other TLR4 agonists, strongly induces Type 1 IFN and T_H_1-biasing chemokines in mouse and human cells [38, 42]. GLA-SE has previously been shown to induce influenza-specific IgG2a titers and ex vivo IFNγ production as an adjuvant with trivalent inactivated flu vaccines [43]. Our studies are the first to combine a recombinant purified subunit RSV sF with GLA-SE, and demonstrate that RSV sF + GLA-SE induced strong neutralizing antibodies and a T_H_1-biased cellular response to RSV in naïve rodents.

Due to the limitations of RSV rodent models, three different rodents were used to characterize RSV-specific adaptive immune responses and protection against RSV challenge. While these challenge studies only used the RSV A2 strain, evaluation of additional strains of RSV may be of interest for future studies. The BALB/c mouse is only semi-permissive for RSV replication and complete protection against RSV challenge is observed even with very low amounts (0.3 μg) of unadjuvanted RSV sF. However, BALB/c mice allow extensive immunological characterization including antibody isotyping and CD4/CD8 T cell cytokine responses against defined RSV F epitopes. Immune responses in BALB/c mice demonstrated that sF + GLA-SE enhances F-specific IgG2a titers and induces T_H_1-biased F-specific CD4 T cells as well as polyfunctional F-specific CD8 T cells that produced IFNγ, TNFα, and IL-2. The cotton rat is more permissive for RSV replication although detailed characterization of the RSV specific immune response is less readily available due to lack of cotton rat specific immunological reagents. Immune responses in cotton rats demonstrated that sF + GLA-SE induces significantly higher RSV neutralizing titers and cellular F-specific IFNγ production than other tested formulations and provides complete protection from RSV challenge not only in the lungs but also in the upper respiratory tract. Protection of the cotton rat upper respiratory tract may require cellular immunity as well as humoral immunity, as passive antibody transfers have protected cotton rat lung but not the nose [44], and an RSV vaccine that induced humoral immunity was also unable to protect the cotton rat nose [45].

The third rodent model used was the Sprague Dawley rat, which is able to accommodate human clinical doses and is routinely employed for toxicology studies in drug and vaccine development. It has not been used as a RSV challenge model and to our knowledge this is the first report of characterization of RSV-specific immune responses in the Sprague Dawley rat. RSV neutralizing titers in response to live RSV A2 in the Sprague Dawley rat were much higher than those achieved in BALB/c mice and similar to those achieved in cotton rats, suggesting that the Sprague Dawley rat may be more permissive for RSV replication than the BALB/c mouse. Immune responses in Sprague Dawley rats confirmed that potential clinical doses of sF + GLA-SE induced significantly higher RSV neutralizing titers and cellular F-specific IFNγ production compared to unadjuvanted sF.

Intramuscular administration of GLA-SE adjuvanted purified recombinant RSV sF induced both RSV neutralizing titers and T_H_1-type F-specific T cell immunity. The RSV sF used in these studies is primarily in the post-fusion conformation. However, both pre- and post-fusion RSV F conformations contain epitopes that can generate effective cross-neutralizing antibodies [46–48]. In our studies, sF + GLA-SE immunized cotton rats demonstrated serum RSV neutralizing titers of 14.7log_2_ (>1:26,000), much higher titers than reported for protective efficacy [44]. Additionally, pooled sera from sF + GLA-SE immunized mice could neutralize circulating clinical RSV A and RSV B strains (S1 Table.). While neutralizing antibodies can decrease infection, F-specific T cell immunity induced by sF + GLA-SE may also be required to blunt RSV disease manifestations in an already seropositive population. Natural infection with RSV tends to skew towards T_H_2-type and away from T_H_1-type immune responses, even more so in older populations that suffer from increased RSV disease burden [49]. The role of T_H_17-type immune responses in RSV infection is still unclear, depending on the experimental system, as IL-17 may negatively modulate T_H_1-type lung responses and viral control [50] or negatively modulate T_H_2-type inflammation [51]. In our models, RSV sF + GLA-SE primarily induces T_H_1-type immune responses. We hypothesize that sF + GLA-SE may boost both humoral and T_H_1-type responses in older adults similar to the boosting of humoral and T_H_1-type responses in older adults observed with trivalent inactivated flu vaccine adjuvanted with GLA-SE ([52] and personal communication, Steve Reed).

Clinical trials of RSV vaccines in the older adult population have thus far focused solely on boosting serological responses such as neutralizing antibody titers. Two intramuscular protein subunit vaccines based on RSV F proteins purified from RSV infected cell lysates have been evaluated in clinical trials in the elderly. Immunization with full length RSV A2 virus-derived purified F protein (PFP-2, ∼98% F protein, 50 μg per dose) generated a 4-fold rise in RSV neutralizing titers in >50% of recipients [53]. A second vaccine using 100 μg of purified viral extract including F, G, and M (matrix) proteins induced a 4 fold rise of RSV neutralizing titers in >50% of elderly recipients [54]. However, no advantage of including alum adjuvant was seen with this vaccine [54]. Interim reports on the immunogenicity of an Sf9 (insect cell)-produced RSV F subunit vaccine in human subjects 60 years and older show only a slight trend to better serological responses in alum adjuvanted compared to unadjuvanted groups [55]. These observations of modest clinical serological responses provide proof of concept for an F protein based intramuscular vaccine in the older adult population, but suggest that alum may not be an optimal adjuvant.

We believe a vaccine that boosts waning T cell responses as well as boosting neutralizing titers could result in an effective RSV vaccine for older adults. Virus-specific T cell memory responses decline with a half-life of 8–15 years [56], much faster than antibody responses. The elderly have T cell defects in RSV responsiveness not seen in the young [10, 11], and despite having similar neutralizing antibody titers to young adults [57], are more susceptible to RSV disease following infection. Aged mice also have defective RSV-specific T cells compared to younger mice and reduced protection from RSV challenge, but vaccine strategies that enhance T cell activity in aged mice improve RSV protection [58]. While neutralizing antibodies are the first line of defense against RSV infection and are a key response to any RSV vaccine, these observations suggests that T cell based immune mechanisms are also important in the control of RSV disease. Declining innate immune responses may decrease adjuvant responsiveness in the elderly, but TLR4 agonists have been shown to improve responses to influenza vaccines in both aged mice and aged humans [52, 59]. Induction of a strong RSV-directed T_H_1 response by a GLA-SE adjuvanted RSV sF vaccine may be able to counteract the T_H_2 bias seen in the elderly and at the same time boost both neutralizing antibodies and T cell responses required to protect against RSV disease. Ultimately the performance of sF + GLA-SE vaccines must be determined in humans, and a Phase 1 study in older (>60 year old) adults is evaluating induction of RSV neutralizing antibodies and T cell responses in addition to safety.

## Supporting Information

S1 Fig RSV sF titration protection study. Mice (N = 5 per group) were immunized at days 0 and 14 with the indicated vaccines and challenged with 6 log_10_ PFU of RSV A2 at day 28. Residual virus in the lungs of animals 4 days post challenge was quantified by plaque assay. Individual results are presented in log_10_PFU/gram, along with a bar representing the group geometric mean and a dotted line indicating the assay LOD, ∼1.4 log_10_ PFU/gram. Individuals with undetectable titers were scored at the LOD.
(TIF)

S2 Fig Total F-specific IgG at Day 28. Mice (N = 7 per group) were immunized at days 0 and 14 with the indicated vaccines. Day 28 sera were evaluated for F-specific IgG by endpoint titer ELISA. Data is presented as the log_2_ reciprocal serum endpoint dilution with a LOD of 5.64. Shown are individual data points, along with a bar representing the group geometric mean with 95% confidence interval. Individuals with undetectable titers were scored at the LOD.
(TIF)

S3 Fig Splenic F-specific CD8 T cell intracellular cytokine staining. Mice were immunized with the indicated RSV sF (0.3 μg) vaccine formulations at days 0 and 14 or with live RSV at day 0 and challenged with 6 log_10_ PFU of RSV at day 28. Spleens were harvested 4 days post challenge (n = 3 for each group) and restimulated 6 hours with an RSV F-derived H-2K^d^ restricted peptide. Cells were surface stained for CD3 and CD8, intracellularly stained for IFNγ, TNFα, and IL-2, and analyzed on an LSR2 for the frequency of responding CD8 T cells. The group mean is shown.
(TIF)

S4 Fig Splenic F-specific IL-4 responses in cotton rats. Cotton rats were immunized with the indicated RSV sF (0.3 μg) vaccine formulations at days 0 and 21 or with live RSV at day 0 and challenged with 6 log_10_ PFU of RSV at day 42. Spleens were harvested 4 days post challenge (n = 4–5 for each group) and restimulated with either media or with RSV sF protein in an IL-4 ELISPOT. F-specific responses were quantified by subtracting the media control values from the test values. (A) Individual IL-4 values are shown with a line representing the group mean. (B) The average ratio of IFNγ to IL-4 spots for each group is shown, with error bars representing the standard error of the mean.
(TIF)

S5 Fig RSV titers in Sprague Dawley rats. Sprague Dawley rats were challenged with 6 log_10_ PFU of RSV A2. Residual virus in the lungs of animals at the indicated timepoints post challenge was quantified by plaque assay (n = 5 per timepoint). Individual results are presented in PFU/gram, along with a bar representing the group geometric mean and a dotted line indicating the highest assay LOD, 4.0 PFU/gram.
(TIF)

S1 Table Cross-neutralization of clinical RSV A and RSV B strains, in log_2_ serum dilution for 50% viral reduction. (DOCX)
