# Supplementary figures and images for: A Novel Respiratory Syncytial Virus (RSV) F Subunit Vaccine Adjuvanted with GLA-SE Elicits Robust Protective TH1-Type Humoral and Cellular Immunity In Rodent Models
Source: PLoS One. 2015 Mar 20;10(3):e0119509. doi: 10.1371/journal.pone.0119509 (PMC4368639; doi:10.1371/journal.pone.0119509)

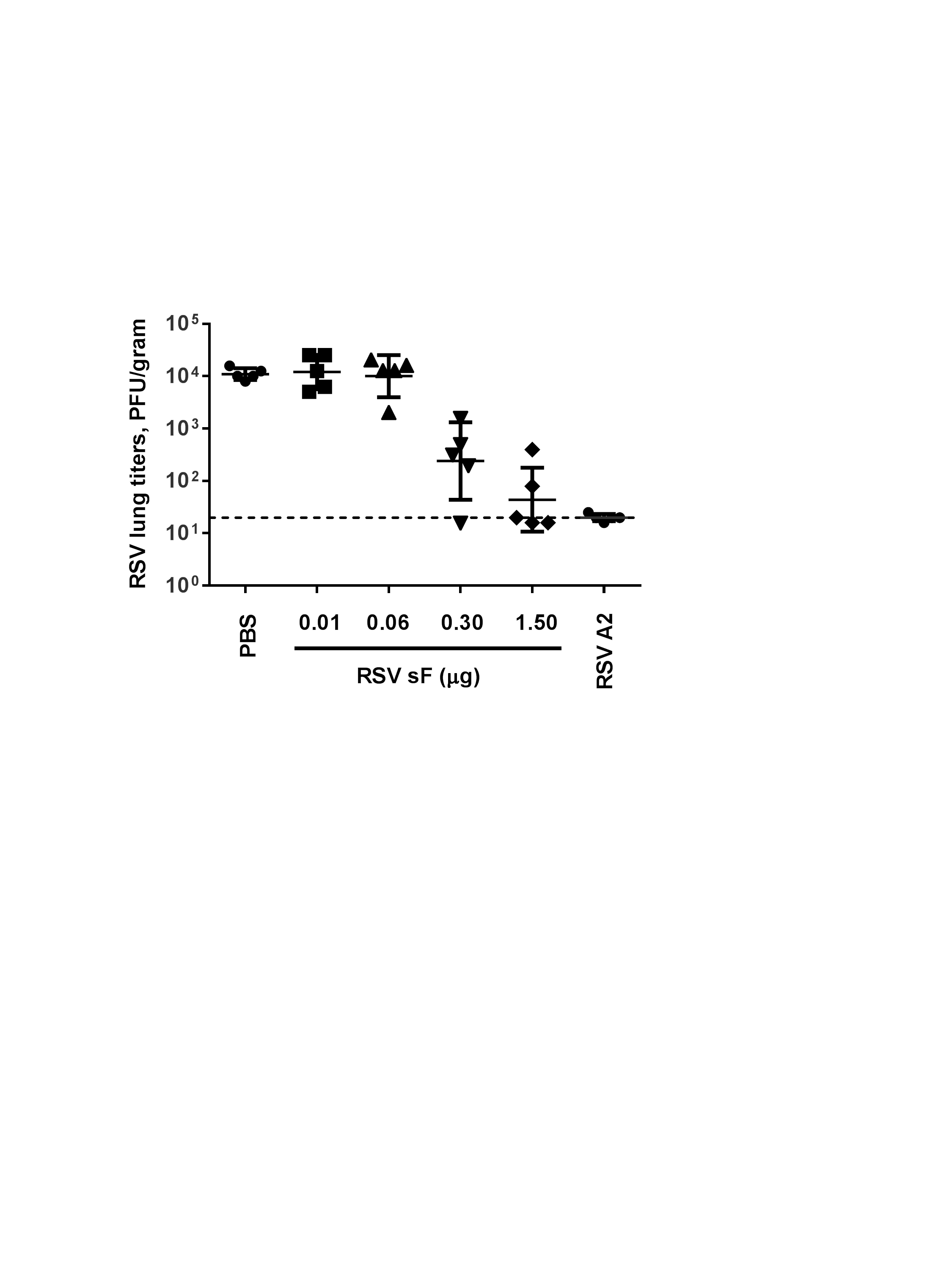

Supplement: S1 Fig — Mice (N = 5 per group) were immunized at days 0 and 14 with the indicated vaccines and challenged with 6 log10 PFU of RSV A2 at day 28. Residual virus in the lungs of animals 4 days post challenge was quantified by plaque assay. Individual results are presented in log10PFU/gram, along with a bar representing the group geometric mean and a dotted line indicating the assay LOD, ∼1.4 log10 PFU/gram. Individuals with undetectable titers were scored at the LOD. (TIF) [file pone.0119509.s001.TIF]

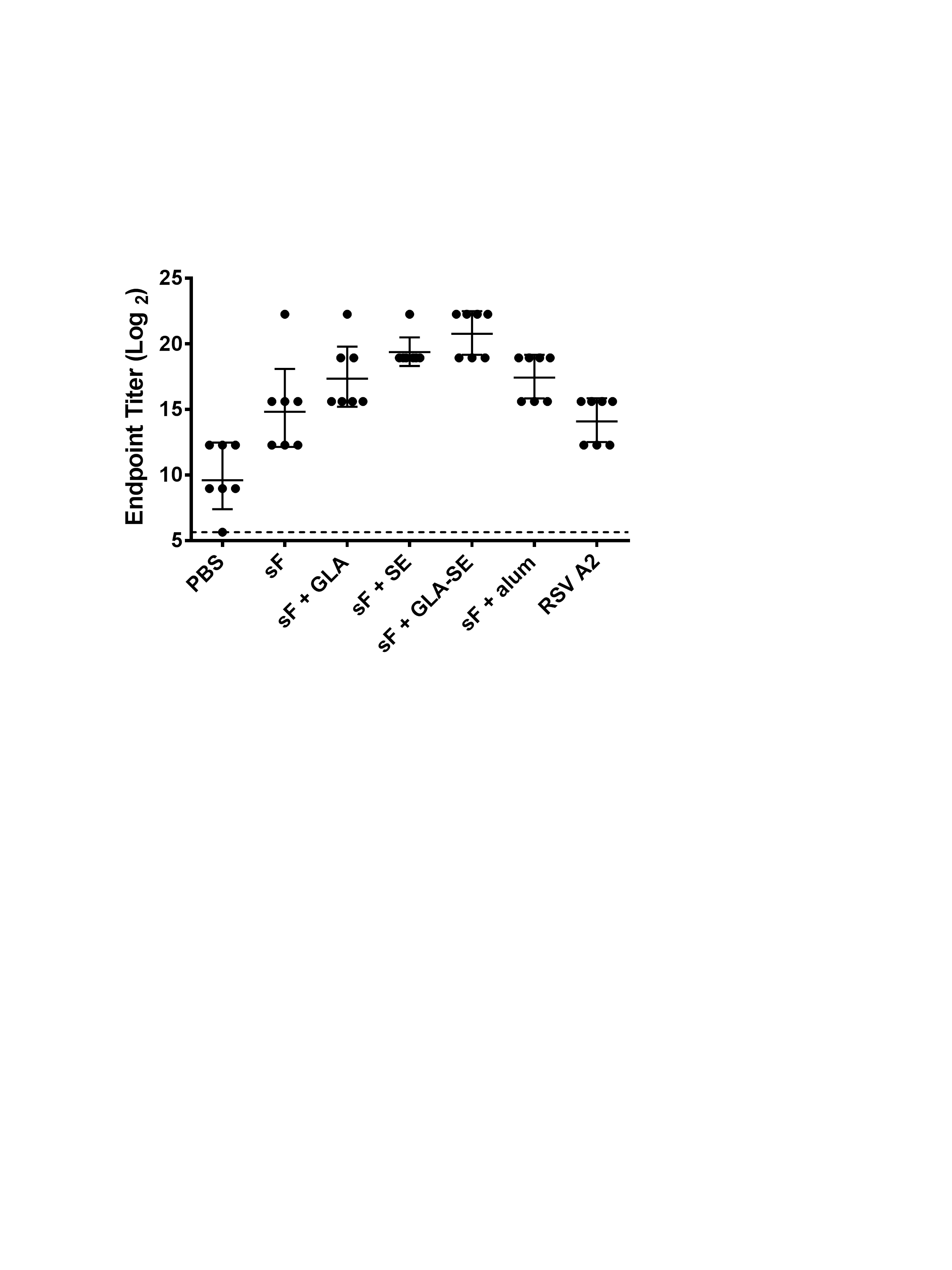

Supplement: S2 Fig — Mice (N = 7 per group) were immunized at days 0 and 14 with the indicated vaccines. Day 28 sera were evaluated for F-specific IgG by endpoint titer ELISA. Data is presented as the log2 reciprocal serum endpoint dilution with a LOD of 5.64. Shown are individual data points, along with a bar representing the group geometric mean with 95% confidence interval. Individuals with undetectable titers were scored at the LOD. (TIF) [file pone.0119509.s002.TIF]

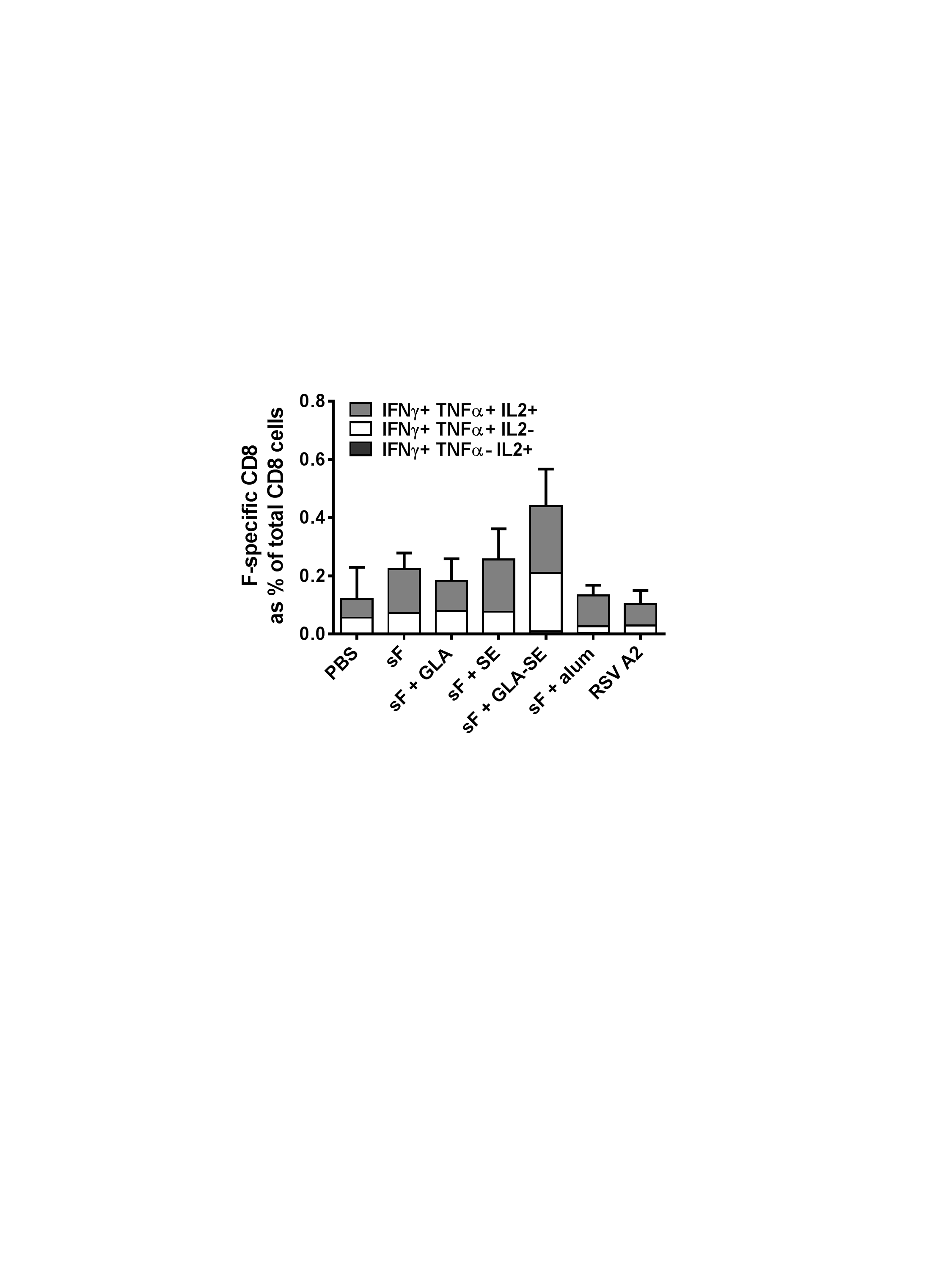

Supplement: S3 Fig — Mice were immunized with the indicated RSV sF (0.3 μg) vaccine formulations at days 0 and 14 or with live RSV at day 0 and challenged with 6 log10 PFU of RSV at day 28. Spleens were harvested 4 days post challenge (n = 3 for each group) and restimulated 6 hours with an RSV F-derived H-2Kd restricted peptide. Cells were surface stained for CD3 and CD8, intracellularly stained for IFNγ, TNFα, and IL-2, and analyzed on an LSR2 for the frequency of responding CD8 T cells. The group mean is shown. (TIF) [file pone.0119509.s003.tif]

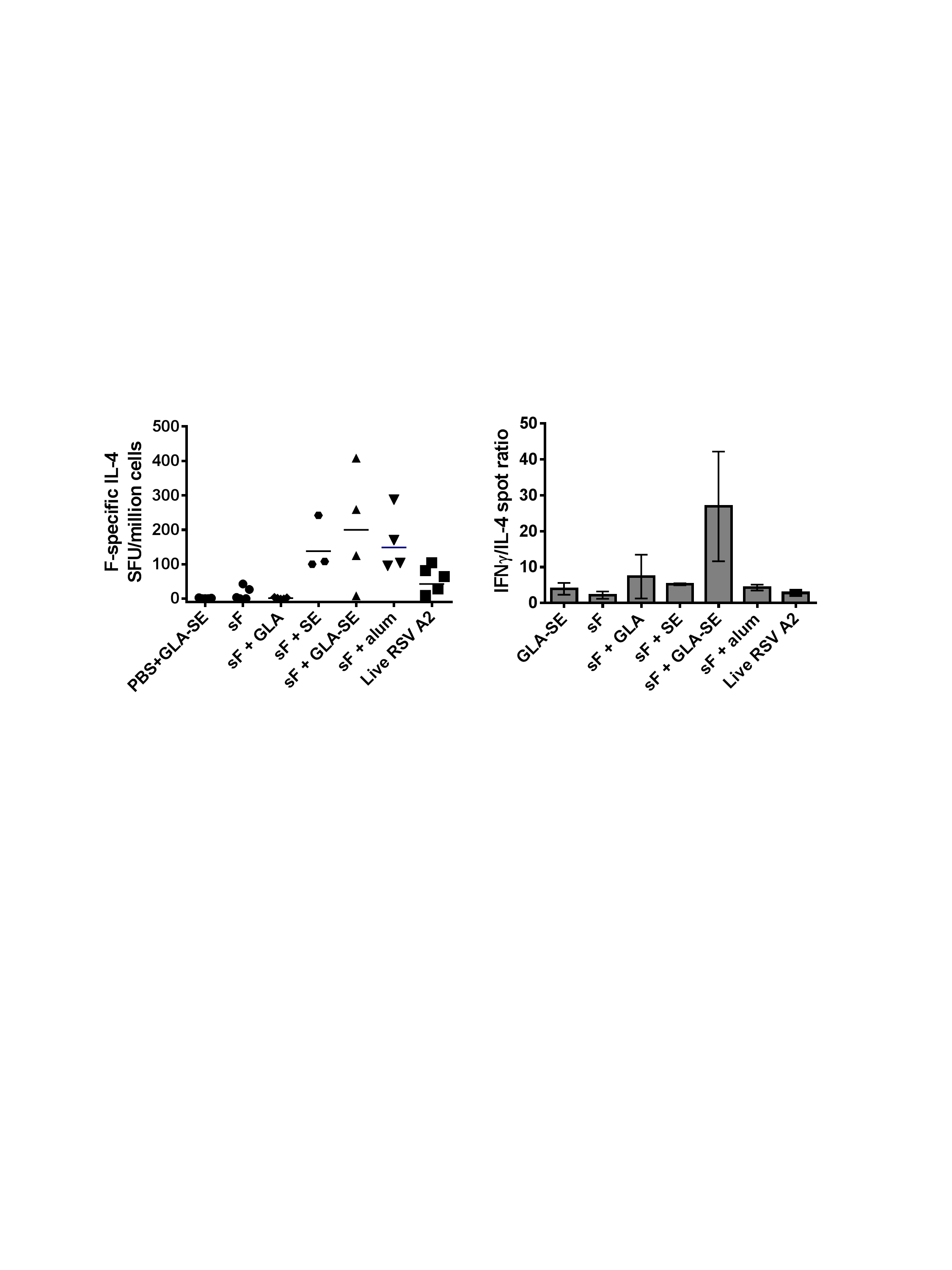

Supplement: S4 Fig — Cotton rats were immunized with the indicated RSV sF (0.3 μg) vaccine formulations at days 0 and 21 or with live RSV at day 0 and challenged with 6 log10 PFU of RSV at day 42. Spleens were harvested 4 days post challenge (n = 4–5 for each group) and restimulated with either media or with RSV sF protein in an IL-4 ELISPOT. F-specific responses were quantified by subtracting the media control values from the test values. (A) Individual IL-4 values are shown with a line representing the group mean. (B) The average ratio of IFNγ to IL-4 spots for each group is shown, with error bars representing the standard error of the mean. (TIF) [file pone.0119509.s004.tif]

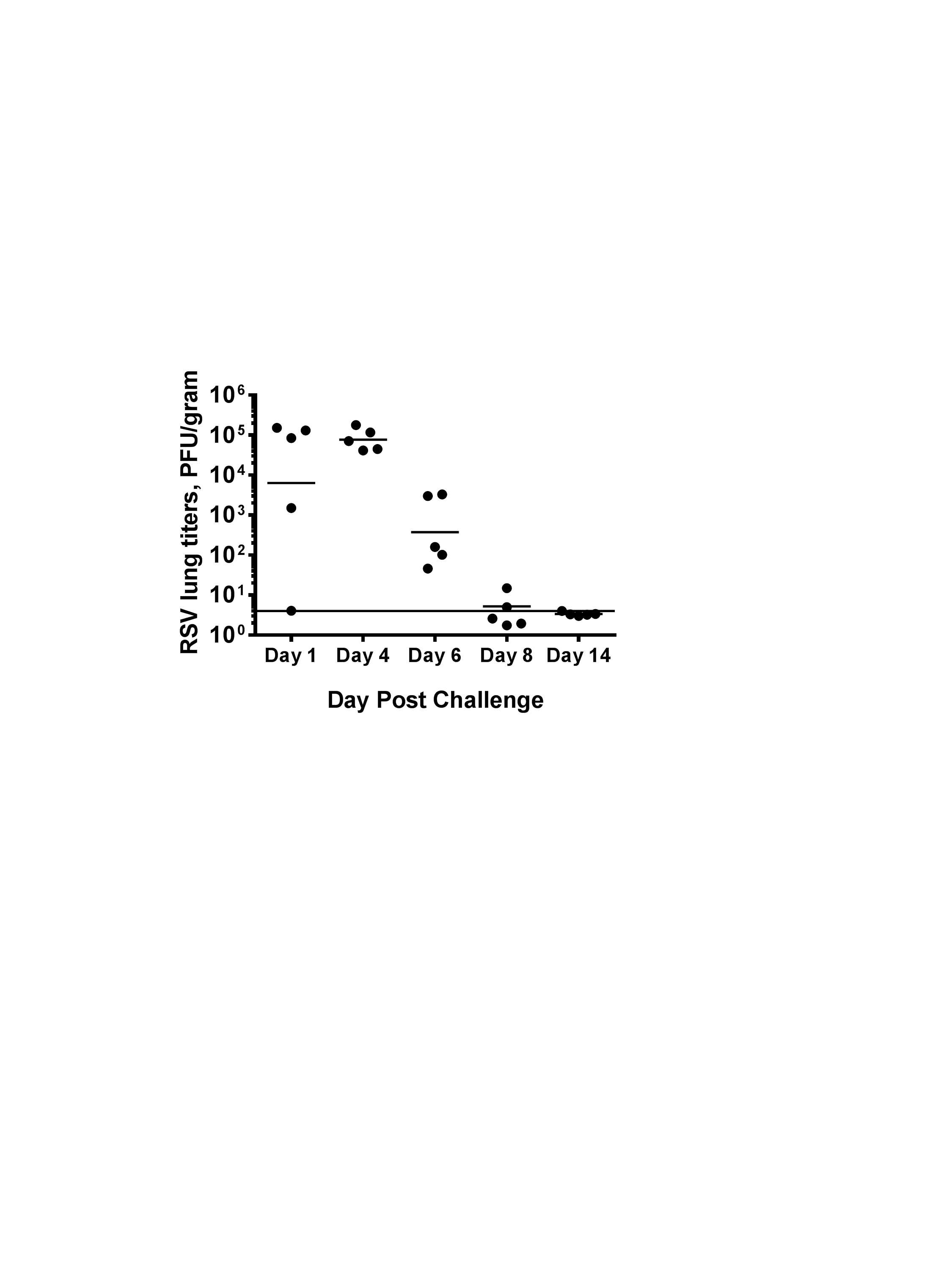

Supplement: S5 Fig — Sprague Dawley rats were challenged with 6 log10 PFU of RSV A2. Residual virus in the lungs of animals at the indicated timepoints post challenge was quantified by plaque assay (n = 5 per timepoint). Individual results are presented in PFU/gram, along with a bar representing the group geometric mean and a dotted line indicating the highest assay LOD, 4.0 PFU/gram. (TIF) [file pone.0119509.s005.tif]
