# Supplementary material for: A Novel Respiratory Syncytial Virus (RSV) F Subunit Vaccine Adjuvanted with GLA-SE Elicits Robust Protective TH1-Type Humoral and Cellular Immunity In Rodent Models
Source: PLoS One. 2015 Mar 20;10(3):e0119509. doi: 10.1371/journal.pone.0119509 (PMC4368639; doi:10.1371/journal.pone.0119509)
Supplement: S1 Table — (DOCX) [file pone.0119509.s006.docx]

**S1 Table. Cross-neutralization of clinical RSV A and RSV B strains, in log_2_ serum dilution for 50% viral reduction.**

| **Virus ^a^** | **sF + GLA-SE pooled immune sera** | **PBS pooled immune sera ^b^** |
| --- | --- | --- |
| RSVA (A2-GFP) | 9.3 | <4.3 |
| RSVA M96-33 | 9.3 | <4.3 |
| RSVA M96-76 | 13.3 | <4.3 |
| RSVA CA-8 | 9.3 | <4.3 |
| RSVB NW VB051507 | 9.3 | <4.3 |
| RSVB 15 | 11.3 | <4.3 |

^a^ Virus detected in micro-neutralization assay modified by staining fixed cells with antibody MAB8262 followed by Alexa488 labeled anti-mouse IgG.

^b^ Results less than the assay limit of detection of 4.3log_2_ are designated as <4.3.
